# Memristive Cluster Based Compact High-Density Nonvolatile Memory Design and Application for Image Storage

**DOI:** 10.3390/mi13060844

**Published:** 2022-05-28

**Authors:** Jingru Sun, Meiqi Jiang, Qi Zhou, Chunhua Wang, Yichuang Sun

**Affiliations:** 1 College of Computer Science and Electronic Engineering, Hunan University, Changsha 410082, China; jt_sunjr@hnu.edu.cn (J.S.); meiqij@hnu.edu.cn (M.J.); qizhou2@hnu.edu.cn (Q.Z.); 2 School of Engineering and Computer Science, University of Hertfordshire, Hatfield AL10 9AB, UK; y.sun@herts.ac.uk

**Keywords:** memristor, memristive cluster, nonvolatile memory, image storage

## Abstract

As a new type of nonvolatile device, the memristor has become one of the most promising technologies for designing a new generation of high-density memory. In this paper, a 4-bit high-density nonvolatile memory based on a memristor is designed and applied to image storage. Firstly, a memristor cluster structure consisting of a transistor and four memristors is designed. Furthermore, the memristor cluster is used as a memory cell in the crossbar array structure to realize the memory design. In addition, when the designed non-volatile memory is applied to gray scale image storage, only two memory cells are needed for the storage of one pixel. Through the Pspice circuit simulation, the results show that compared with the state-of-the-art technology, the memory designed in this paper has better storage density and read–write speed. When it is applied to image storage, it achieves the effect of no distortion and fast storage.

## 1. Introduction

With the scale of CMOS on the nanometer scale, the technological progress predicted by Moore’s Law is more and more difficult to meet, which limits the rapid development of information technology. The memristor [1,2] as a new device after resistance, capacitance, and inductance, has been widely used in the design of neural networks [2,3,4,5,6,7,8,9,10], logic circuits [11,12,13,14,15], and chaotic circuits [16,17,18,19,20,21,22,23,24,25,26,27] because of its characteristics of low power consumption, low floor area, device scalability, nonvolatile, fast switching speed, high switching durability, and CMOS compatibility. Especially with the explosive growth of data and the increasingly serious storage bottleneck problem in the era of big data, a new type of memory with high storage density and fast read and write speed is urgently needed. Memristors as nonvolatile devices are considered to be one of the most promising candidate devices, which has aroused great interest.

The memristor was first raised by Chua in 1971, indicating a relationship between flux and charge. Then, in 2008, HP Lab presented a physical model of a two-terminal electrical device that behaves like a perfect memristor. Since then, researchers have manufactured various physical models which are made of a variety of materials [28,29] and show different characteristics. Meanwhile, to fit different memristor devices, people have proposed different models, like linear ion drift, the Simmons tunneling model, ThrEshold Adaptive Memristor (TEAM) model [30], Voltage ThrEshold Adaptive Memristor (VTEAM) model [31], Drift Speed Adaptive Memristor (DSAM) model [32], etc.

Early memristor-based memory designs employ the internal state of the memristor to achieve multi-level storage, but the circuit structure was complex and the density of memory is low [33]. In 2018, Mohammad et al. proposed a faster and stable 6T1M memristor memory cell [34]; however, the memory cell has a more complex structure and lower storage density. In 2014, Zangeneh et al. proposed a 1T1R memristor memory with crossbar array [35], and then the 1T1M [36] structure was proposed. In this structure, the transistor is a switch to make the memory cell unidirectional to avoid sneak path current problems. The 1T1M structure is widely used because of its stability and high storage density. In 2018, Wang et al. proposed a 1T2M [37] structure memristor memory with crossbar array, which greatly increases the storage density, but this structure has two word lines, which increases the complexity of the circuit and brings new sneak path current problem. Then, Sun et al. [38] proposed a new 1T2M structure, which solves the sneak path current problem with one word line. Some scholars have tried to use all-memristor designs to realize the design of memory cells, such as the structure of 2M1M [39] and 2M2M [40], 2M is used to replace the transistor to realize the switching function, and avoid sneak path current, all-memristor designs increase the storage density, but the problem of sneak path current is inevitable. Therefore, how to improve the storage density while solving the sneak path current problem is the research focus of memristor memory.

In our previous works [38,40], an extensible memristive cluster structure enabling multi-value memory is proposed, which can improve the store density by adding memristors with different characteristics. On the basis of this work, this paper proposes a 4M memristive cluster, which is connected with a transistor to form a memory cell of a crossbar array, to realize a 4-bit multi-valued nonvolatile memory. Furthermore, we apply the 4-bit multi-valued nonvolatile memory for image storage. Simulation results show that the proposed multi-value memory has the characteristics of high reading/writing speed, high store density and low power consumption, and can realize stable storage. The memristive cluster structure provides a new scheme for the design of memory and logic circuits, which can be applied to digital image storage, vector data storage, and logic circuit design, providing stable multi-value storage functions.

This paper is organized as follows: In Section 1, the background of memristive memory is presented. In Section 2, the DSAM memristor model used in this paper is introduced. In Section 3, based on the DSAM memristor model, a memristor cluster, memory cell, and cross array circuit are designed. In Section 4, the memory circuit designed above is applied to image storage. Section 5 is the simulation part. Through Pspice software, we obtain the simulation results of the voltage-current relationship of the DSAM memristor model and the memory cell, the read–write operation, read–write time, time delay and power consumption of the memory cell, the storage density, and sneak path current of the cross array circuit, and analyze the difference between the stored image and the original image. Finally, Section 6 concludes this paper.

## 2. DSAM Model

Because the resistance of the DSAM model memristor will change only when the voltage reaches the threshold value, and it has the advantages of fast change speed and stable maintenance after reaching the state. With its good binary characteristic, we chose the DSAM model to describe the memristor and simulation in this paper, and its *i*-*v* relationship is

(1) $$v\left(t\right)=R_{off}-x△R\cdot i\left(t\right),$$

where the state variable x represents the normalized broadband of the conductive area, and its range is [0,1]. In addition, △$R=R_{off}-R_{on}$, $R_{off}$, and $R_{on}$ are the maximum resistance and minimum resistance of the memristor. Additionally, the corresponding state variable is x=1 and x=0. Therefore, the derivative of the state variable x can be expressed as follows

(2) $$\frac{dx}{dt}=\left\{\begin{array}{cc} & k_{on}\cdot ▵R\cdot i\left(t\right)\cdot f\left(x\right),\;\;\;\;\;\;v\left(t\right)>v_{th,}\\ & 0,\;\;\;\;\;\;\;\;\;\;\;\;\;\;\;\;\;\;\;\;\;\;\;\;\;\;\;\;\;\;\;\;\;\;\;\;\;\;\;\;\;\;\;\;\;\;\;\;\;\;\;\;\;\;\;-v_{th}\leq v\left(t\right)\leq v_{th,}\\ & k_{off}\cdot ▵R\cdot i\left(t\right)\cdot f\left(x\right),\;\;\;\;v\left(t\right)<-v_{th.} \end{array}\right.$$

where

(3) $$f\left(x\right)=\left\{\begin{array}{cc} & a\cdot \;{\left(1-x\right)}^{p},\;\;\;\;\;\;\;\;\;\;\;\;v(t)>0,\\ & \left(a\cdot x\right)\;{}^{p}\;,\;\;\;\;\;\;\;\;\;\;\;\;\;\;\;\;\;\;\;\;v(t)\leq 0. \end{array}\right.$$

**Figure 1:**
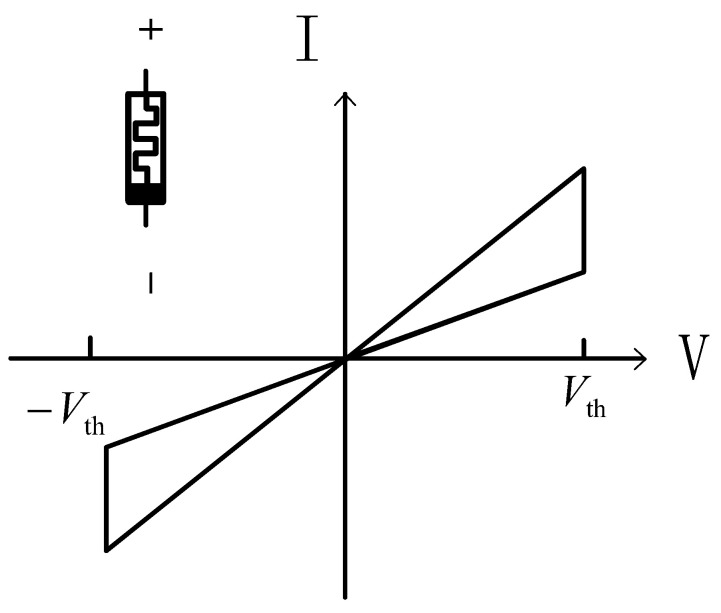
The relationship between voltage and current of the memristor.

Among them, $v_{th}$ and $-v_{th}$ represent positive and negative threshold voltages, *a* and *p* are curve fitting parameters, and $k_{on}$ and $k_{off}$ are linear adjustable parameters. Only when the input applied voltage meets the threshold condition ($v_{th}$), the state of the memristor will be changed. The relationship between voltage and current of the memristor can be described in Figure 1.

## 3. A Memristive Cluster Based 4-Bit Crossbar Array Memory

### 3.1. Memristive Cluster

**Figure 2:**
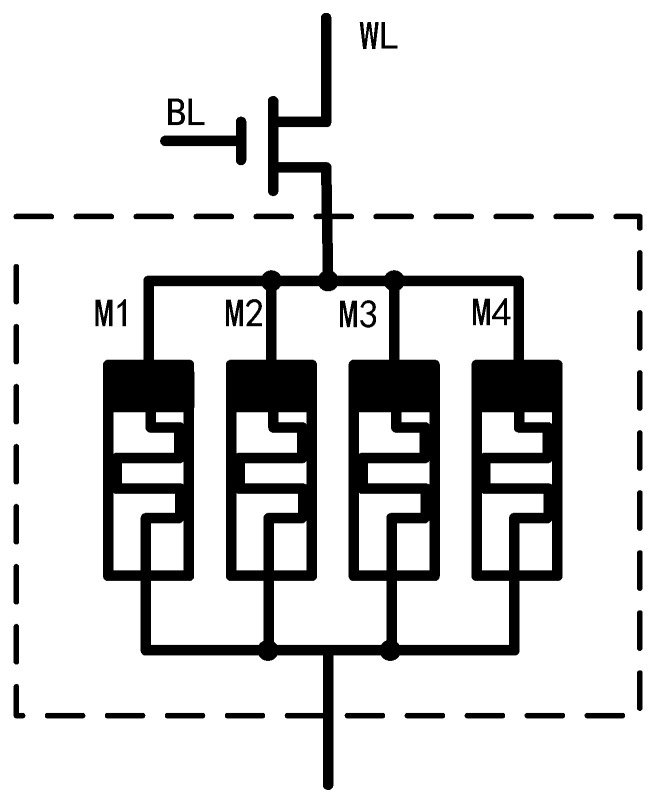
A 1T4M memory cell.

Based on the DSAM model mentioned above, we propose a memristive cluster structure consisting of four memristors, as shown in the dotted line in Figure 2. The four memristors have different threshold voltages and threshold resistances. Assume that the threshold voltages and resistances of the four memristors satisfies

(4) $$R_{on4}=2\cdot R_{on3}=4\cdot R_{on2}=8\cdot R_{on1}=8\cdot R_{on},$$

(5) $$R_{off4}=R_{off3}=R_{off2}=R_{off1}=R_{off},$$

where $R_{off}\gg R_{on}$.

### 3.2. 1T4M Nonvolatile Memory Cell

Based on the memristive cluster, we propose a non-volatile memory cell consisting of a transistor and a memristive cluster, as shown in Figure 2. The transistor works as a switch, four memristors are connected in parallel and in series with the transistor. We can realize storage by controlling the voltage on each memristor, thus changing the state of each memrister.

Suppose $R_{on}$ and $R_{off}$ represent logic 1 and 0 respectively, the logic value of $M_{4}$ is the lowest bit of the 4-bit binary, and the $M_{1}$ logic value of is the highest. Thus, the total resistance of memory cell $R_{c}=\frac{1}{\frac{1}{R_{M1}}+\frac{1}{R_{M2}}+\frac{1}{R_{M3}}+\frac{1}{R_{M4}}}$. Thus, we can know the corresponding logic values of the memory cell according to the different total resistance. Different logic values correspond to different memory cell resistance values, and the resistance values are distinguished. When we apply the same read voltage, there is a visible distinction between the currents through the memory cell. Therefore, this memory cell can stably achieve 4-bit storage. Each memory memristor has two resistive states, $R_{on}$ and $R_{off}$ and the memory cell has corresponding 16 logical values as shown in Table 1.

**Figure 3:**
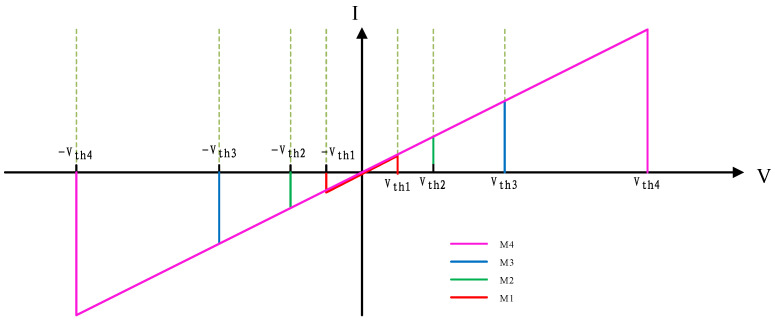
The relationship between voltage and current of $M_{1}$, $M_{2}$, $M_{3}$, and $M_{4}$.

When a sinusoidal voltage is applied to four memristors, the current-voltage characteristic curves of $M_{1}$, $M_{2}$, $M_{3}$, and $M_{4}$ perform as illustrated in Figure 3, having conspicuous threshold features.

Assume that the initial states of $M_{1}$, $M_{2}$, $M_{3}$, and $M_{4}$ are all $R_{off}$. Therefore, the resistance state of the memristor will change only when we give a positive voltage. When the voltage across memory cell $V_{S}=V_{1}$, the resistances of memristors remain the same since $V_{S}$ < $V_{th1}$. When $V_{S}$ changes to $V_{2}$, the resistance of $M_{1}$ changes to $R_{on}$ and others unchanged. When $V_{S}$ gets into the range of $V_{3}$, there are two memristors, $M_{1}$ and $M_{2}$, whose resistance values changed to be $R_{on}$ and $R_{on2}$. When $V_{S}=V_{4}$, the resistances of memristors are $R_{on}$, $R_{on2}$, $R_{on3}$, and $R_{off}$, respectively. When $V_{S}=V_{5}$, the resistances of memristors all changes to $R_{on}$, $R_{on2}$, $R_{on3}$, and $R_{on4}$. However when the previous state of memristor is $R_{on}$, we need to apply negative voltage to change its resistance state. The relationship between the resistance values of $M_{1},M_{2},M_{3}$, and $M_{4}$ and the voltage range is shown in the Table 2. The $R_{pre}$ represents the resistance of the memristor in the previous state.

#### 3.2.1. Write Operation

Suppose $R_{on}$ and $R_{off}$ represent logic 1 and 0, the logic value of memristor $M_{4}$ is the lowest bit of the 4-bit binary, the logic value of $M_{1}$ is the highest. The initial state of all memristors in this paper is $R_{off}$. Thus, the logic value of the memory cell is “0000”. To write correct data to the memory cell, we should set appropriate voltage impulses across the memristors to change their states. The operations of writing data to the memory cell are as follows.

Operation 1:

Write logic value “1110”. It requires one step. Set write voltage $V_{S}=V_{4}$, because $V_{S}$ locates in the range of $V_{4}$, which is higher than $V_{th1}$, $V_{th2}$, and $V_{th3}$, expect $V_{th4}$, so the states of $M_{1}$, $M_{2}$, and $M_{3}$ change to $R_{on}$ and $M_{4}$ remains. Thus, the logic value of the memory cell is “1110”.

Operation 2:

Write logic value “0110”. It requires two steps. Firstly, set write voltage $V_{S}=V_{4}$, as the operation 1, the logic value of the memory cell is “1110”. Secondly, set write voltage $V_{S}=-V_{2}$, which is only higher $M_{1}$. So the logic value of $M_{1}$ converts to 0, but $M_{2}$, $M_{3}$, and $M_{4}$ are unchanged. Then the logic value of the memory cell is “0110”.

Operation 3:

Write logic value “1101”. It requires three steps. Firstly, set write voltage $V_{S}=V_{5}$. Because $V_{5}$ is higher than each threshold voltage, so the states of all memristors change to $R_{on}$, logic 1. The logic value of the memory cell is “1111”. Secondly, set $V_{S}=-V_{4}$. The logic values of $M_{1}$, $M_{2}$, and $M_{3}$ convert to 0, but $M_{4}$ remains unchanged, logic 1. Then the logic value of the memory cell is “0001”. Lastly, set write voltage $V_{S}=V_{3}$. The logic values of $M_{1}$ and $M_{2}$ change to logic 1, but others remain unchanged. Therefore, the finally logic value of the memory cell is “1101”.

**Figure 4:**
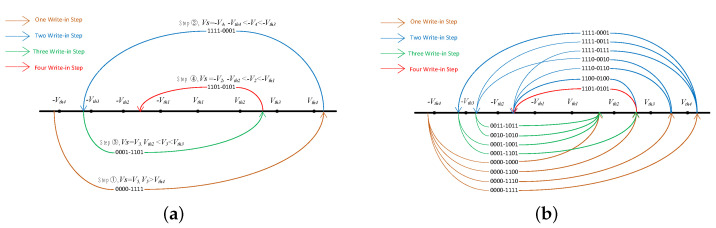
The step of writing data 1010 (**a**) and other steps of 16 kinds of 4-bit data (**b**).

Operation 4:

Write logic value “0101”. It requires four steps. Firstly, set write voltage $V_{S}=V_{5}$. Because $V_{5}$ is higher than each threshold voltage, the logic values of all memristors change to 1. So the total logic value of the memory cell is “1111”. Secondly, set $V_{S}=-V_{4}$. The logic values of memristors $M_{1}$, $M_{2}$, and $M_{3}$ convert to 0, but $M_{4}$ remains unchanged, logic 1. Then the logic value of the memory cell is “0001”. Thirdly, set $V_{S}=V_{3}$. The logic values of memristors $M_{1}$ and $M_{2}$ change to 1, but others remain unchanged. Then the logic value of the memory cell is “1101”. Lastly, set $V_{S}=V_{2}$. The logic value of $M_{1}$ converts to 0, but others remain unchanged. Therefore, the finally logic value of the memory cell is “0101”. Figure 4a shows the specific steps of writing data 1010.

As it can be seen from the above operations, the steps required to store different 4-bit binary data in the memory cell are different. Figure 4b shows the steps to write 16 types of 4-bit data. When writing information by writing operation, some information can be completed in one step, while others even need four steps. In Figure 4, an arc represents a one-step write operation, and the previous number in the middle of the arc refers to the information state stored at this time. By giving the voltage to the arrow at the end of the line segment, the information state stored in the next step is reached. The data stored by multi-step write operation will reach other states first through each step, and then reach the final desired result.

#### 3.2.2. Read Operation

The read operation is usually performed after the write operation. When the data are stored in the memory cell, a read voltage is applied to the memory cell. At this time, each memristor maintains its state after the write operation. By measuring the current value flowing through each memory cell and comparing it with the comparison current, the value stored in the memory cell at this time can be read.

When the stored values are different, the total conductivity value and the current value after pressurization of the memory cell are shown in Table 3.

### 3.3. Memristor Crossbar Array Design

**Figure 5:**
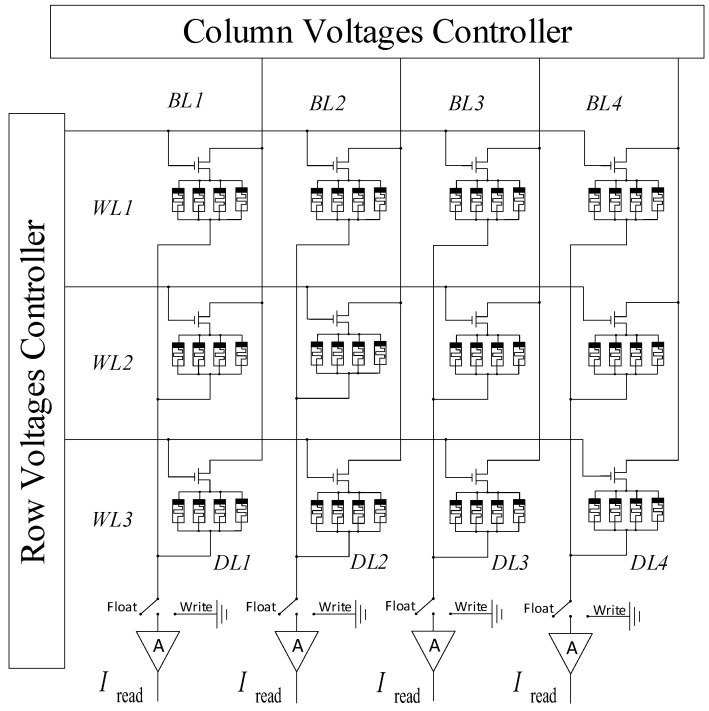
A 3 × 4 crossbar array architecture design.

When the proposed memory cells apply to the binary or ternary memory, the crossbar of multi-valued storage is shown in Figure 5. The memory cells in the same row can be written and read parallelly. For example, the memory cells A, B, C, and D can be activated and have the write operation by bit lines (BL1, BL2, BL3, BL4). Then, we give them a read voltage impulse to read the current by the DL1, DL2, DL3, and DL4. DL1, DL2, DL3, and DL4 are usually connected to an amplifier. Thus, we can attain the data of memory cells A, B, C, and D in parallel by DL1, DL2, DL3, and DL4, respectively.

## 4. Memristor Crossbar Array in Image Storage

In this paper, two kinds of resistance states of memristors are used, assuming that they represent 0 and 1 respectively. By changing the voltage value at both ends of the memory cell, the resistance value can be changed to achieve the purpose of storing data. Through simulation and experiments, it is verified that by providing different pulse voltages, the four memristors can reach sixteen different states, and each state can represent a four-bit binary number. Memristor has the advantages of small size, fast reading and writing speed, low power consumption, and non-volatility, so this paper proposes to use it for image storage. The concrete implementation method is as follows.

Firstly, because the memristor stores binary values, the pixel information of the image should be extracted when storing the image, converted into binary information, and then stored. In the actual simulation experiment, this paper selects two images and extracts the pixel information of the image through MATLAB. Because the value of each pixel of the gray scale image is 0–255, each pixel can be represented by an eight-bit binary number. As mentioned earlier, one memory cell can store 4-bit information, so two memory cells are used to store one pixel value in this paper. Due to the limitation of simulation software, the memristor crossbar array built in this paper is composed of 8 × 16 memory cells, which can store 8 × 8 pixel information at a time. In fact, more information can be stored at a time.

Then, the pixel information extracted before is written into the memory cell by writing operation, and the resistance state of the memristor is in one-to-one correspondence with pixel information 1 and 0 by providing different pulse voltages so that the image pixel values are stored in the memory cell.

Finally, the current value of each memory cell is read out through the read operation. Compared with the current threshold we set, it is restored to the corresponding 4-bit binary number. An 8-bit binary number obtained from the two memory cells is a pixel.

## 5. Simulation

**Figure 6:**
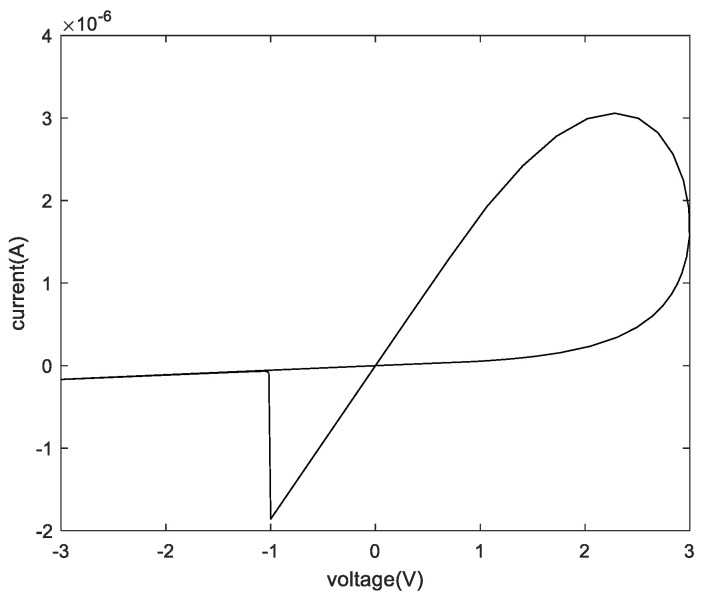
The relationship between voltage and current of one memristor.

**Figure 7:**
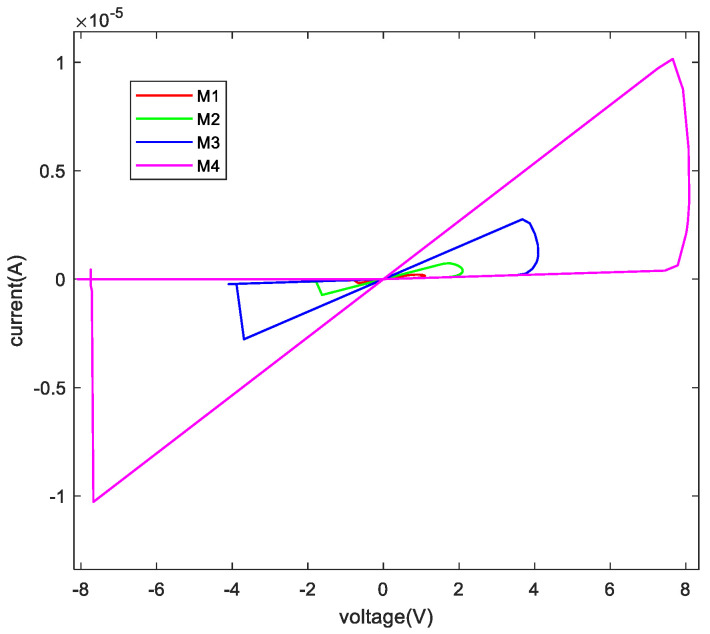
The relationship between voltage and current of the four memristors.

**Figure 8:**
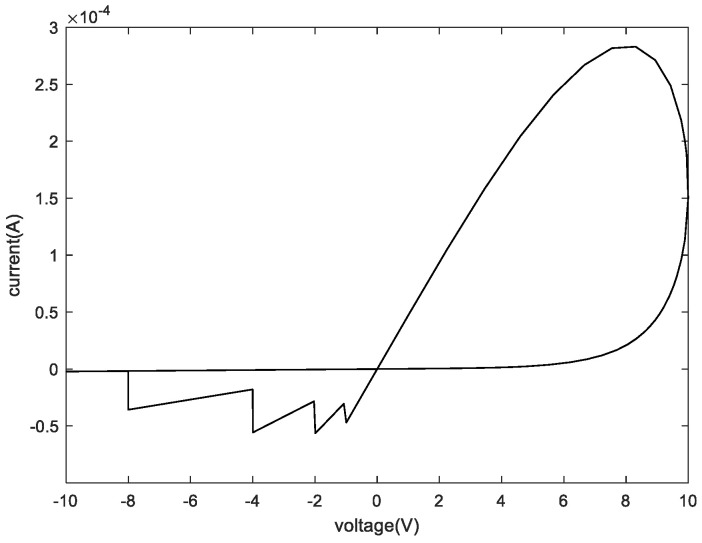
The relationship between voltage and current of the memory cell.

The memristor model used in this paper is the DSAM model. The specific parameters of this paper when using the DSAM model for simulation are shown in Table 4. Through the simulation of a single memristor and a 1T4M memory cell, the voltage and the current characteristic curve are shown in the following figures. Figure 6 shows the voltage and the current characteristic curve of one memristor, Figure 7 shows the voltage and current characteristic curve of the four different memristors, and Figure 8 shows and current characteristic curve of the memory cell.

### 5.1. Write Simulation

The write operation is to change the state of the memristor by changing the voltage to realize the storage of data. The following are the Pspice simulation results of the specific written data.

**Figure 9:**
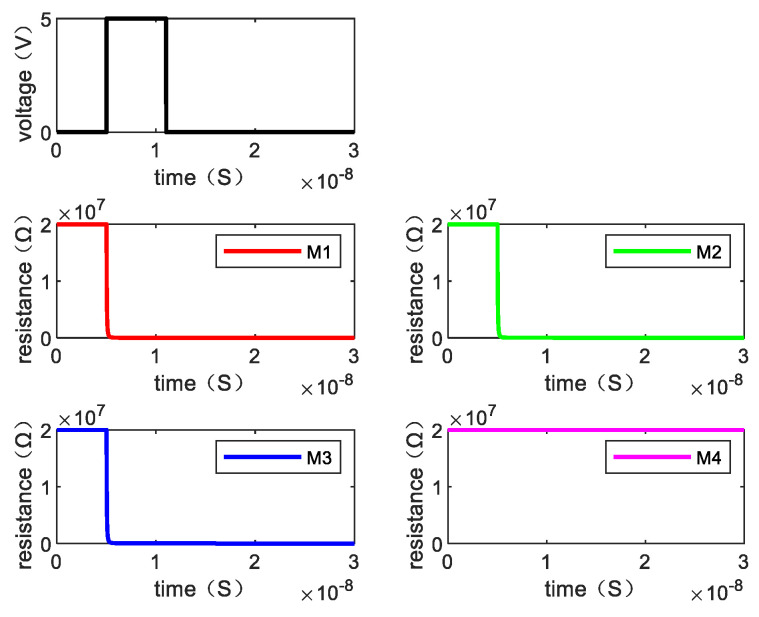
The resistance value of each memristor changes when writing “1110”.

**Figure 10:**
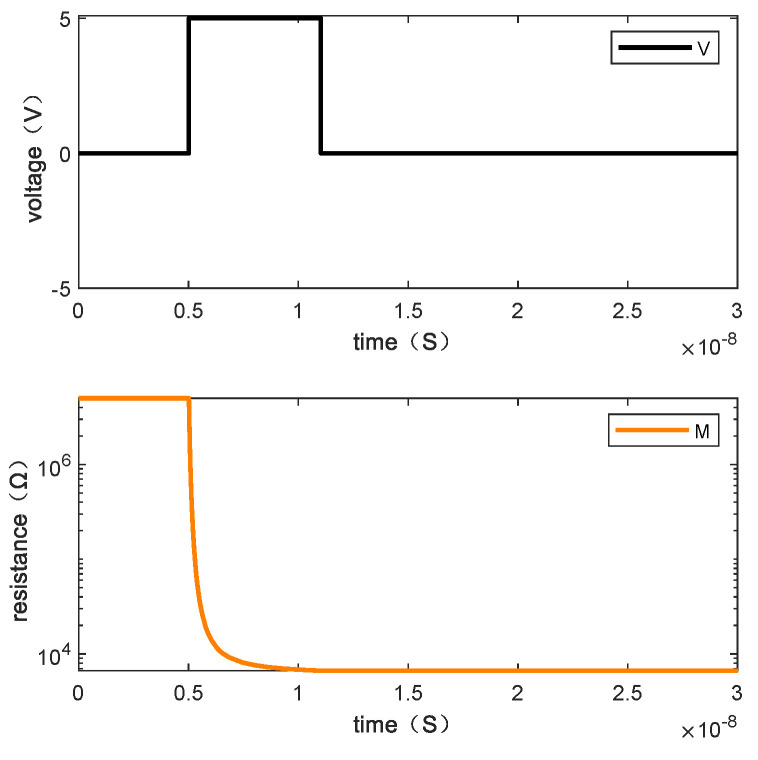
The changes of resistance value of the memory cell when writing “1110”.

Writing “1110” requires one step. Figure 9 shows the resistance changes of memristor $M_{1}$, $M_{2}$, $M_{3}$, and $M_{4}$ when the written data is “1110” by giving the voltage and Figure 10 is the resistance change of the memory cell.

**Figure 11:**
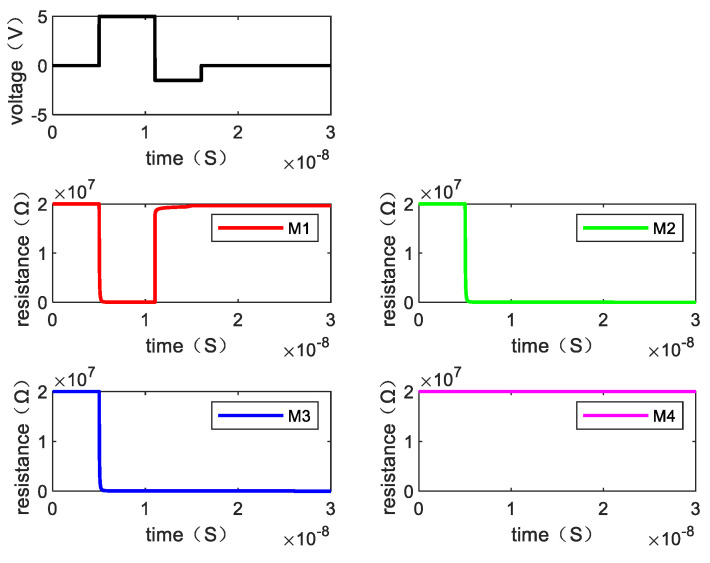
The resistance value of each memristor changes when writing “0110”.

**Figure 12:**
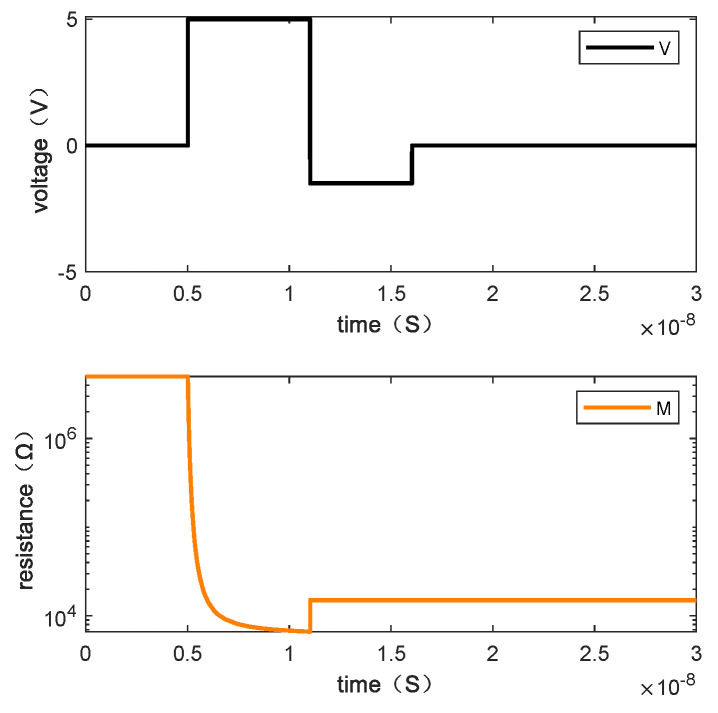
The changes of resistance value of the memory cell when writing “0110”.

Writing “0110” requires two steps. Figure 11 shows the resistance changes of memristor $M_{1}$, $M_{2}$, $M_{3}$, and $M_{4}$ when the written data is “0110” by giving the voltage and Figure 12 is the resistance change of the memory cell.

**Figure 13:**
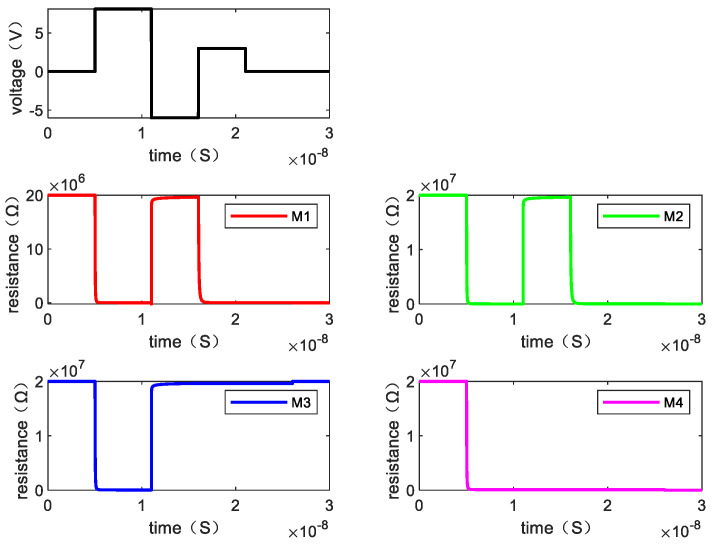
The changes of resistance value of each memristor when writing “1101”.

**Figure 14:**
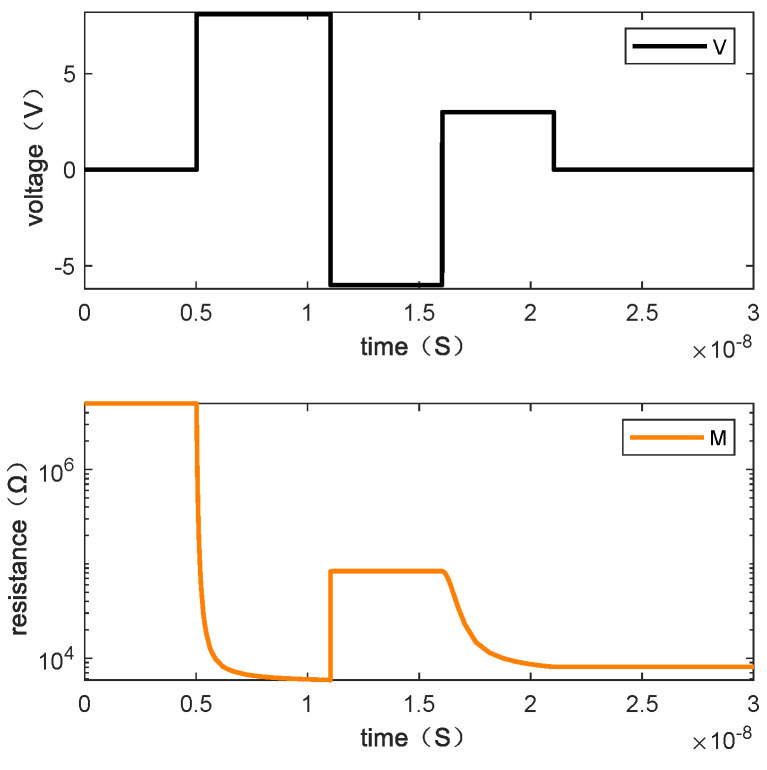
The changes of resistance value of the memory cell when writing “1101”.

Writing “1101” requires three steps. Figure 13 shows the resistance changes of memristor $M_{1}$, $M_{2}$, $M_{3}$, and $M_{4}$ when the written data is “1101” by giving the voltage and Figure 14 is the resistance changes of the memory cell.

**Figure 15:**
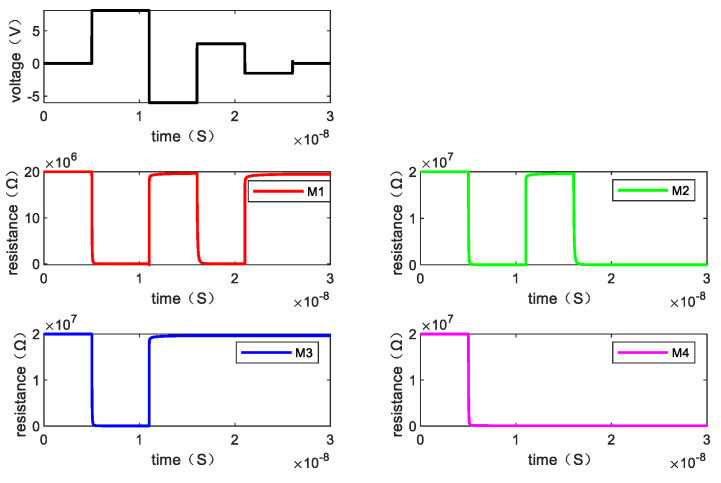
The changes of resistance value of each memristor when writing “0101”.

**Figure 16:**
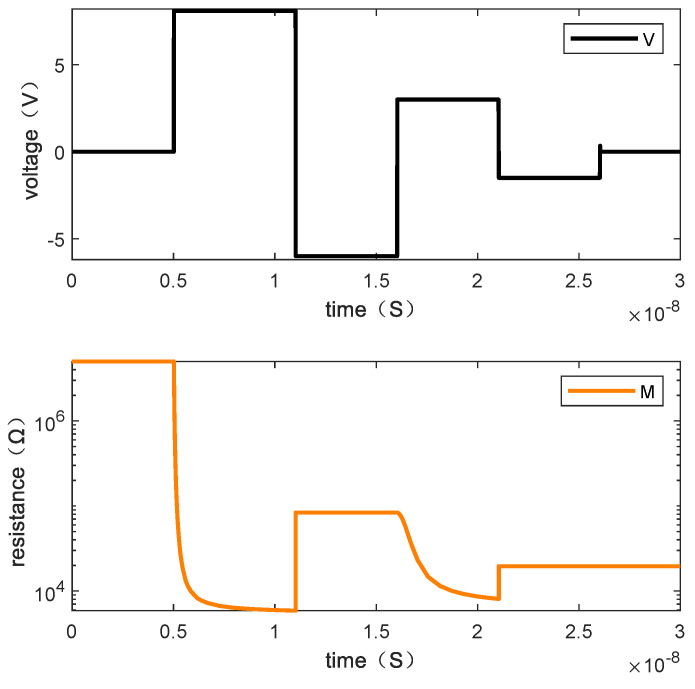
The changes of resistance value of the memory cell when writing “0101”.

Writing “0101” requires four steps. Figure 15 shows the resistance changes of memristor $M_{1}$, $M_{2}$, $M_{3}$, and $M_{4}$ when the written data is “0101” by giving the voltage and Figure 16 is the resistance changes of the memory cell.

**Figure 17:**
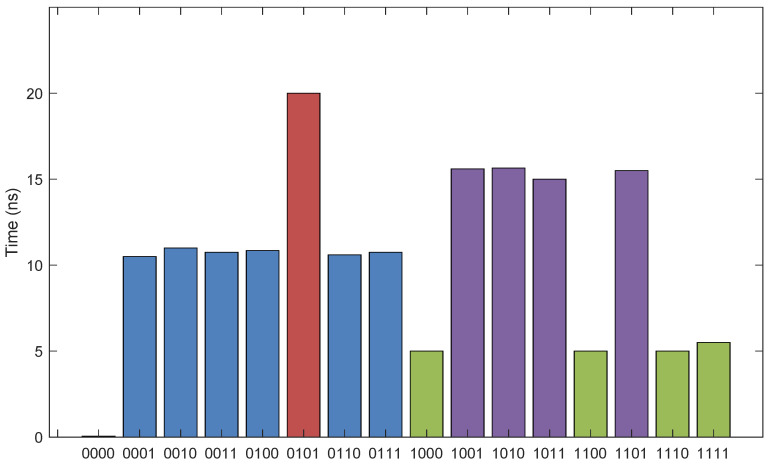
The time of the write operation.

Due to the different steps of writing data, the time of writing operation is also different. The duration of the pulse voltage supplied in each step designed in this paper is 5 ns, and the delay of each step operation is about 0.01 ns. The actual time required for writing data is shown in Figure 17, and the average time for writing a datum is about 10 ns.

### 5.2. Read Simulation

**Figure 18:**
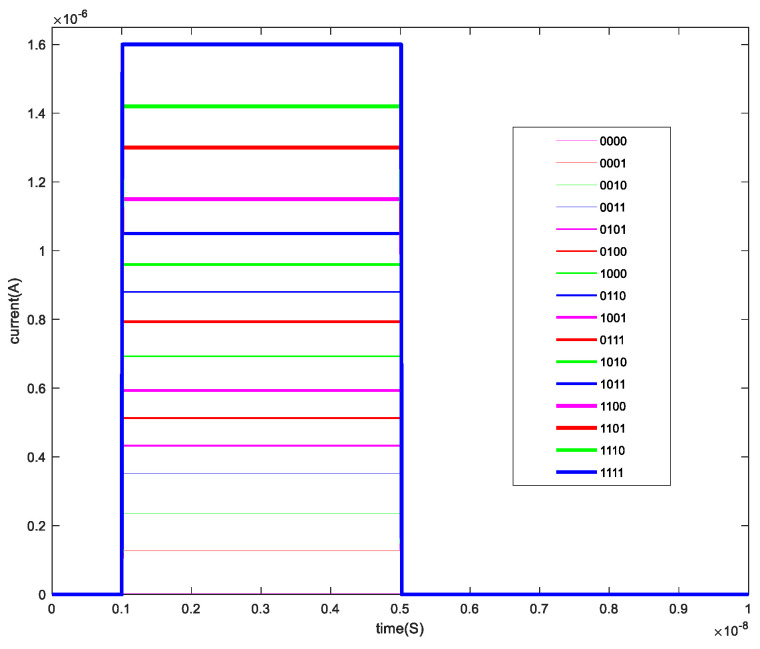
The current of the read operation.

The state of each memristor in the memory cell is changed through the write operation. On this basis, the read operation is to apply an applied voltage to the memory cell and read out the output current after passing through the unit. Figure 18 shows the output current when writing data “0000”, “0001”, “0010”, “0011”, “0100”, “0101”, “0110”, “0111”, “1000”, “1001”, “1010”, “1011”, “1100”, “1101”, “1110”, and “1111”. At this time, the applied voltage is 0.01 V.

**Figure 19:**
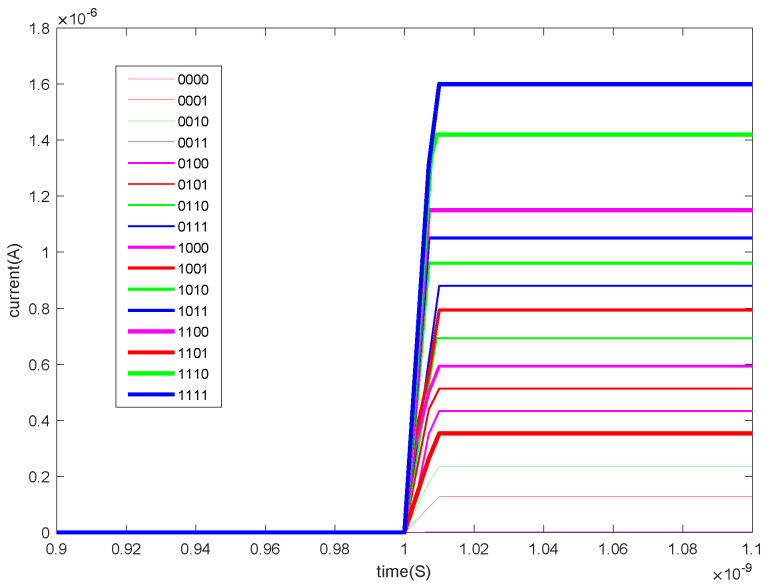
The time delay of the read operation.

The time delay of the read operation can be seen in Figure 19. When the read voltage is 0.01 V, the delay of the read operation is about 0.01 ns.

### 5.3. Energy Consumption Simulation

**Figure 20:**
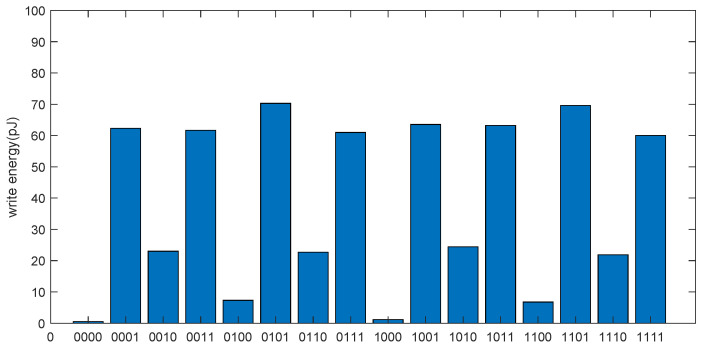
The energy consumption of 16 kinds of write operations.

Different logic values are written through the write operation, and the energy consumption of 16 write operations is simulated. The simulation results are shown in Figure 20. Due to the different write steps and write voltages of different logic values, the energy consumption will be affected.

### 5.4. Image Storage Simulation

Based on the above research, this paper proposes to apply it to image storage. Using the binary characteristics of the memristor, the collected image information is transformed into binary numbers, which are saved in the array circuit through a write operation, and then read out the information through a reading operation to recover the image.

**Figure 21:**
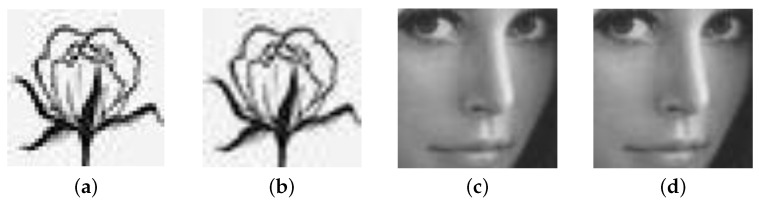
The original image of (**a**) flower and (**c**) lena, the stored image of (**b**) flower and (**d**) lena.

Figure 21 illustrates the simulation in image storage. Figure 21a is an image of a flower drawn by MATLAB with a resolution of 40 × 40, and the pixel information can be obtained by extracting through MATLAB are 40 × 40 8-bit binary numbers. A memory cell can store a 4-bit binary number, and this paper builds a circuit with 32 × 16 1T4M cells structure through Pspice.

The information is stored in this circuit by applying a pulse voltage to each memory cell. After the information is stored, a reading voltage is applied, we can read out the currents value and the corresponding binary number is determined according to the size of the current value, and then restored through MATLAB. Figure 21b is the restored image of Figure 21a after storage.

Figure 21c,d show the simulation results of a Lena image with pixel information of 64 × 64 respectively.

To evaluate the quality of the restored image, the correlation coefficient between the stored image *G* and the original image *T* can be calculated by

(6) $$r_{TG}=\frac{E(T-E(T))(G-E(G))}{\sqrt{D(T)D(G)}}$$

where D(T) and D(G) are the square deviations of the restored image and the original image, respectively, $D\left(x\right)=\frac{1}{N}$·$\sum_{N}^{i=1}{\left(x_{i}-E\left(X\right)\right)}^{2}$ and $E\left(x\right)=\frac{1}{N}$·$\sum_{N}^{i=1}x_{i}$. Theoretically, $r_{TG}\leq 1$, and the larger the correlation coefficient, the better the imaging effect. Through this method, we can get the restored image without information loss after storage.

In the circuit designed in this paper, only two memory cells are needed to save a pixel of a gray scale image. Nowadays, vector data is widely used, and this 4-bit storage technology will bring faster reading and writing speed. It is more suitable for the design of memory such as artificial intelligence chips, and special memory to store images.

### 5.5. Sneak Path Current Analysis

The memristor crossbar array designed in this paper supports parallel data storage. When storing data for each row, the memory cells of other rows are disconnected and there is no sneak path current. Through Pspice simulation, the sneak path current of the same column is very small relative to the read–write current, which can be ignored.

**Figure 22:**
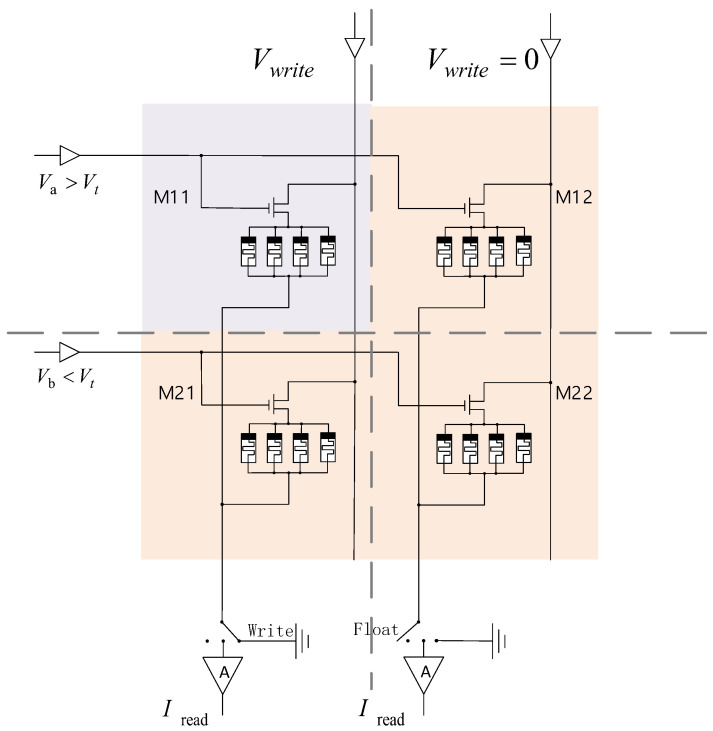
A structure of four adjacent memory cells.

**Figure 23:**
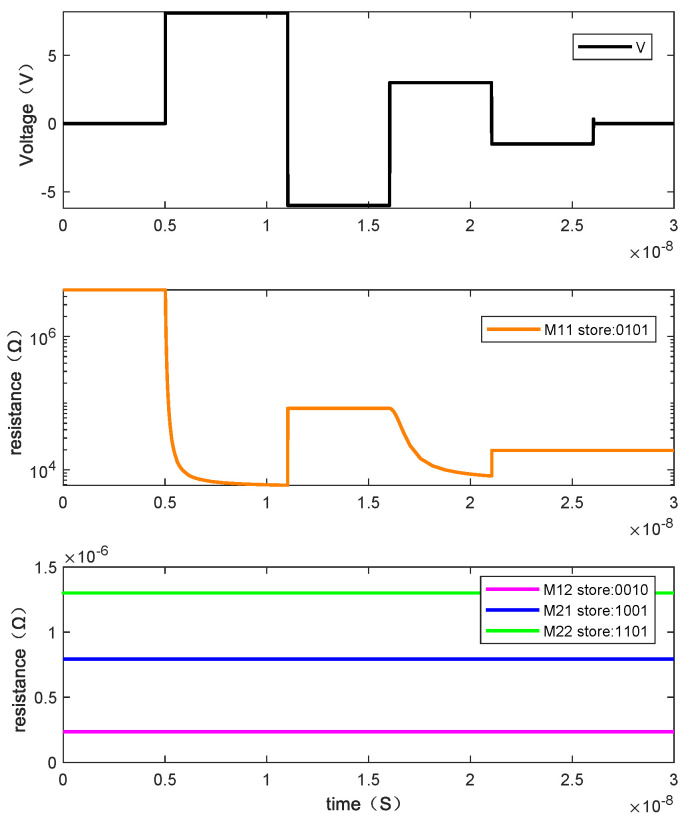
The resistance value changes of the *M*_12_, *M*_21_, and *M*_22_ when writing “0101” to the memory $M_{11}$.

In Figure 22, *M*_11_ is a memory cell that needs to write data, and the information stored in its adjacent memory cells *M*_12_, *M*_21_ and *M*_22_ are “0010”, “1001”, and “1101”, respectively. When writing the datum “0101” to *M*_11_, we can see the change of its resistance value with the voltage, as shown in Figure 23. At this time, it can be found that the resistance values of the three adjacent memory cells remain unchanged, that is, the stored information is unaffected. Therefore, it can be concluded that the write operation on a memory cell will not change the state of the adjacent memristors, that is, it will not be affected by the sneak path current.

Additionally, during the read operation, the sneak path current is also 0 after testing. To sum up, the circuit designed in this paper has no sneak path current in the process of storing and reading data.

### 5.6. Density

Storage density is a key point in storing information. This paper compares it with the existing work, as shown in Table 5. According to the authors of [18,19,20] the density of 1T1M memory cell is 1.6 Gbt/cm^2^, 1T2M is 3.2 Gbt/cm^2^, and 2D1M memory cell is only 1.2 Gbt/cm^2^. In our work, the density of 1T4M can reach 6.5 Gbt/cm^2^, which is significantly higher than other similar structures.

## 6. Conclusions

This paper proposes a 1T4M multi-valued nonvolatile crossbar array memory and applies it to image storage. The memory cell is composed of a 4M memristive cluster and a transistor, which can save 4-bit values, and the transistor effectively mitigates the sneak path current problem. Simulation results show that the storage density of the proposed memory is almost twice the existing CMOS+memristor storage density, 4-bit values can be read in one read operation, the cell in the same rows can be read and written parallel, the cell in different columns can be read and write parallel, and the reading/writing speed is greatly improved. The proposed non-volatile memory is used to save gray scale images and achieves good stability, with only two memory cells per pixel. This memory has the property of high storage density, quick reading/writing speed, and low power, and is suitable for storage of vector-matrix structured data. In addition, the memristive cluster structure has scalability and richer dynamic properties, which can be applied to the design of memristive neural networks, which is also our next work.

## Figures and Tables

**Table 1:** Sixteen logical values corresponding to the memory cell.

*M*_1_	*M*_2_	*M*_3_	*M*_4_	Logic Value
$R_{off}$	$R_{off}$	$R_{off}$	$R_{off}$	0000
$R_{off}$	$R_{off}$	$R_{off}$	$R_{on4}$	0001
$R_{off}$	$R_{off}$	$R_{on3}$	$R_{off}$	0010
$R_{off}$	$R_{off}$	$R_{on3}$	$R_{on4}$	0011
$R_{off}$	$R_{on2}$	$R_{off}$	$R_{off}$	0100
$R_{off}$	$R_{on2}$	$R_{off}$	$R_{on4}$	0101
$R_{off}$	$R_{on2}$	$R_{on3}$	$R_{off}$	0110
$R_{on}$	$R_{on2}$	$R_{on3}$	$R_{on4}$	0111
$R_{on}$	$R_{off}$	$R_{off}$	$R_{off}$	1000
$R_{on}$	$R_{off}$	$R_{off}$	$R_{on4}$	1001
$R_{on}$	$R_{off}$	$R_{on3}$	$R_{off}$	1010
$R_{on}$	$R_{off}$	$R_{on3}$	$R_{on4}$	1011
$R_{on}$	$R_{on2}$	$R_{off}$	$R_{off}$	1100
$R_{on}$	$R_{on2}$	$R_{off}$	$R_{on4}$	1101
$R_{on}$	$R_{on2}$	$R_{on3}$	$R_{off}$	1110
$R_{on}$	$R_{on2}$	$R_{on3}$	$R_{on4}$	1111

**Table 2:** The relationship between the resistance values of $M_{1},M_{2},M_{3}$, and $M_{4}$ and the voltage range.

	$V_{S}$	$R_{M1}$	$R_{M2}$	$R_{M3}$	$R_{M4}$
$-V_{5}$	$\left(-\infty ,-V_{th4}\right)$	$R_{off}$	$R_{off}$	$R_{off}$	$R_{off}$
$-V_{4}$	$\left(-V_{th4},-V_{th3}\right)$	$R_{off}$	$R_{off}$	$R_{off}$	$R_{pre}$
$-V_{3}$	$\left(-V_{th3},-V_{th2}\right)$	$R_{off}$	$R_{off}$	$R_{pre}$	$R_{pre}$
$-V_{2}$	$\left(-V_{th2},-V_{th1}\right)$	$R_{off}$	$R_{pre}$	$R_{pre}$	$R_{pre}$
$V_{1}$	$\left(-V_{th1},V_{th1}\right)$	$R_{pre}$	$R_{pre}$	$R_{pre}$	$R_{pre}$
$V_{2}$	$\left(V_{th1},V_{th2}\right)$	$R_{on}$	$R_{pre}$	$R_{pre}$	$R_{pre}$
$V_{3}$	$\left(V_{th2},V_{th3}\right)$	$R_{on}$	$R_{on2}$	$R_{pre}$	$R_{pre}$
$V_{4}$	$\left(V_{th3},V_{th4}\right)$	$R_{on}$	$R_{on2}$	$R_{on3}$	$R_{pre}$
$V_{5}$	$\left(V_{th4},\infty \right)$	$R_{on}$	$R_{on2}$	$R_{on3}$	$R_{on4}$

**Table 3:** The total conductivity value and the current value after pressurization of the memory cell.

$M_{1}$	$M_{2}$	$M_{3}$	$M_{4}$	Logic Value	G	I
$R_{off}$	$R_{off}$	$R_{off}$	$R_{off}$	0000	$4/R_{off}$	$G\cdot V_{C}$
$R_{off}$	$R_{off}$	$R_{off}$	$8\cdot R_{on}$	0001	$\left(24R_{on}+R_{off}\right)/8\cdot R_{on}R_{off}$	$G\cdot V_{C}$
$R_{off}$	$R_{off}$	$4\cdot R_{on}$	$R_{off}$	0010	$\left(12R_{on}+R_{off}\right)/4\cdot R_{on}R_{off}$	$G\cdot V_{C}$
$R_{off}$	$R_{off}$	$4\cdot R_{on}$	$8\cdot R_{on}$	0011	$\left(16R_{on}+3R_{off}\right)/8\cdot R_{on}R_{off}$	$G\cdot V_{C}$
$R_{off}$	$2\cdot R_{on}$	$R_{off}$	$R_{off}$	0100	$\left(6R_{on}+R_{off}\right)/2\cdot R_{on}R_{off}$	$G\cdot V_{C}$
$R_{off}$	$2\cdot R_{on}$	$R_{off}$	$8\cdot R_{on}$	0101	$\left(16R_{on}+5R_{off}\right)/8\cdot R_{on}R_{off}$	$G\cdot V_{C}$
$R_{off}$	$2\cdot R_{on}$	$4\cdot R_{on}$	$R_{off}$	0110	$\left(8R_{on}+3R_{off}\right)/4\cdot R_{on}R_{off}$	$G\cdot V_{C}$
$R_{off}$	$2\cdot R_{on}$	$4\cdot R_{on}$	$8\cdot R_{on}$	0111	$\left(8R_{on}+7R_{off}\right)/8\cdot R_{on}R_{off}$	$G\cdot V_{C}$
$R_{on}$	$R_{off}$	$R_{off}$	$R_{off}$	1000	$\left(3R_{on}+R_{off}\right)/R_{on}R_{off}$	$G\cdot V_{C}$
$R_{on}$	$R_{off}$	$R_{off}$	$8\cdot R_{on}$	1001	$\left(16R_{on}+9R_{off}\right)/8\cdot R_{on}R_{off}$	$G\cdot V_{C}$
$R_{on}$	$R_{off}$	$4\cdot R_{on}$	$R_{off}$	1010	$\left(8R_{on}+5R_{off}\right)/4\cdot R_{on}R_{off}$	$G\cdot V_{C}$
$R_{on}$	$R_{off}$	$4\cdot R_{on}$	$8\cdot R_{on}$	1011	$\left(8R_{on}+11R_{off}\right)/8\cdot R_{on}R_{off}$	$G\cdot V_{C}$
$R_{on}$	$2\cdot R_{on}$	$R_{off}$	$R_{off}$	1100	$\left(4R_{on}+3R_{off}\right)/2\cdot R_{on}R_{off}$	$G\cdot V_{C}$
$R_{on}$	$2\cdot R_{on}$	$R_{off}$	$8\cdot R_{on}$	1101	$\left(8R_{on}+13R_{off}\right)/8\cdot R_{on}R_{off}$	$G\cdot V_{C}$
$R_{on}$	$2\cdot R_{on}$	$4\cdot R_{on}$	$R_{off}$	1110	$\left(4R_{on}+7R_{off}\right)/4\cdot R_{on}R_{off}$	$G\cdot V_{C}$
$R_{on}$	$2\cdot R_{on}$	$4\cdot R_{on}$	$8\cdot R_{on}$	1111	$15/8R_{on}$	$G\cdot V_{C}$

**Table 4:** The memristor parameters we use in this paper.

	a	p	$K_{on}$	$K_{off}$	$R_{on}$ (KΩ)	$K_{off}$ (MΩ)	$V_{th}\;(V)$	$-V_{th}\;(V)$
*M*_1_	2.1	1.8	8000	5000	10	20	1	−1
*M*_2_	2.1	1.8	8000	5000	20	20	2	−2
*M*_3_	2.1	1.8	8000	5000	40	20	4	−4
*M*_4_	2.1	1.8	8000	5000	80	20	8	−8

**Table 5:** Density.

Designs	1T1M [18]	1T2M [20]	2D1M [22]	1T4M
Density (Gbt/cm^2^)	1.6	3.2	1.2	6.5

## Abbreviations

The explanation of abbreviations:

$R_{on}$	the minimum resistance of the memristor
$R_{off}$	the maximum resistance of the memristor
$R_{oni}$	$R_{on}$ of the ith memristor in the memory cell
$R_{offi}$	$R_{off}$ of the ith memristor in the memory cell
$R_{Mi}$	the resistance of the ith memristor
$R_{pre}$	the resistance of the previous state
$M_{i}$	the ith memristor in the memory cell
$R_{C}$	the total resistance of the memory cell
$V_{S}$	write operation voltage
$V_{thi}$	threshold voltage of the ith memristor
